# Non motor symptoms in progressive supranuclear palsy: prevalence and severity

**DOI:** 10.1038/s41531-017-0037-x

**Published:** 2017-12-08

**Authors:** Fabiana Giada Radicati, Pablo Martinez Martin, Chiara Fossati, Kallol Ray Chaudhuri, Margherita Torti, Carmen Rodriguez Blazquez, Laura Vacca, Fabrizio Stocchi

**Affiliations:** 1 Institute for Research and Medical Care IRCCS San Raffaele, Rome, Italy; 2 Carlos III Institute of Health and CIBERNED, Madrid, Spain; 3 National Parkinson Foundation Centre of Excellence, Kings College, London, UK; 4 Department of Neuro-rehabilitation Sciences, Casa Cura Policlinico (CCP), Milan, Italy

## Abstract

NMSs have been extensively studied in PD patients but not in other forms of parkinsonism such as Progressive Supranuclear Palsy (PSP). The primary objective of this study was to analyze the frequency, severity and the type of non-motor symptoms (NMS) in PSP patients using the non-motor symptoms scale (NMSS). The secondary objective was to differentiate NMS between PSP and Parkinson’s disease (PD). We enrolled in this cross-sectional study 50 consecutive PSP and 100 matched Parkinson’s disease (PD) patients, in the proportion PSP/PD = 1/2, matched in age, sex, and disease duration. Motor and Non Motor symptoms (different scales for each disease) were evaluated at baseline using PSP scale, SCOPA Motor, Montreal Cognitive Assessment (MOCA), HADS, Hamilton, and Non Motor Symptom scale (NMSS). Comparative analysis was done using chi-squared test, Mann-Whitney test and Fisher’s exact test. Fifty PSP (56% female) and 100 PD (59% female) patients completed the study protocol and were included for statistical analysis. The NMSS total domains score in the PSP group was 77.58 ± 42.95 (range 14–163) with NMS burden grade: 4, very severe, and the in the PD group was 41.97 ± 35.45 (range: 0–215) with NMS burden grade: 3, severe. The comparative analysis showed that NMS total score (*p* < 0.0001), Sleep/Fatigue (*p* = 0.0007), Mood/Apathy (*p* = 0.0001), Gastrointestinal (*p* < 0.0001), and Urinary dysfunction (*p* = 0.0001) domains were significantly more severe in PSP patients than in PD. This observational study reports that NMSs are very frequent in PSP patients hence the higher burden of NMS in PSP specifically related to mood/apathy, attention/memory, gastrointestinal, urinary disturbances compared to PD.

## Parkinsonism: understanding the non-motor symptom burden

Non-motor symptoms are more frequent and severe in patients with Progressive Supranuclear Palsy (PSP) than in those with Parkinson’s disease (PD). An international team led by Fabrizio Stocchi at the Institute for Research and Medical Care IRCCS in Rome used the Non-motor Symptoms Scale (NMSS) to compare the symptoms experienced by 50 PSP patients and 100 PD patients. They found that patients with PSP suffered worse and more frequent gastrointestinal and urinary disturbances, as well as more severe dysfunctions in attention and memory compared with PD patients. Moreover, the reported lack of motivation and sleep disturbances were more burdensome in PSP patients. These findings will not only contribute to diagnose PSP earlier and more accurately, but also help to increase awareness of the impact of non-motor symptoms on patients’ quality of life.

## Introduction

Non-dopaminergic and non-motor symptoms (NMSs) are sometimes present before diagnosis of Parkinson’s disease (PD) and atypical or secondary parkinsonism (AP), and almost inevitably emerge with disease progression. Indeed, non-motor symptoms often contribute to severe disability, impaired quality of life, and shortened life expectancy.^1^

NMS have been extensively studied in PD patients but not in other form of parkinsonism such as progressive supranuclear palsy (PSP). Indeed, some studies have reported the frequency of NMS in PSP. In the PRIAMO (PaRkinsondIseAse non-MOtor symptoms) study, 30 patientswith PSP were enrolled, and it was shown that the most prevalent NMS in PSP were gastrointestinal (GI) problems and fatigue. NMS prevalence was much higher in parkinsonism compared to PD, the result being aligned with the rapid and severe course of this rare disease.^2^

In a Korean study on the interrelationship between NMS in atypical Parkinsonism (AP) and PD (117 PD and 57 AP, 12 of them with PSP), the results showed that the most frequent symptoms in PSP were attention/memory problems. They also described thatin the PSP group mood/cognition, attention/memory and gastrointestinal tract symptoms increased faster in the later stages of the disease.^3^

Schmidt et al. established that PSP patients had frequently significant autonomic dysfunction. The parasympathetic cardiovascular system appeared to be involved to a similar extent in PD and PSP patients, while sympathetic cardiovascular dysfunction is more recurrent and critical in PD patients, but can also be found in PSP patients.^4,5^

Two different studies from the NNIPPS (the Natural History and Neuroprotection in Parkinson Plus Syndromes) study group reported that PSP patients showed a higher rate of cognitive, behavioral and urinary dysfunctions.^6,7^

A longitudinal study, where some patients with PSP were also included, indicates a severe and complex medley of non-motor and motor symptoms in the palliative stage.^8^

Despite these studies, the epidemiology and the clinical characteristics of NMS in atypical and secondary parkinsonism are still not well explored.

The objective of this study was to analyze the frequency, gravity and the type of non-motor symptoms in PSP patients using a global and comprehensive scale, the non-motor symptoms scale (NMSS). The secondary objective was to differentiate NMS between PSP and Parkinson’s disease (PD).

## Results

50 PSP (56% female) and 100 PD (59% female) completed the study protocol and were included for statistical analysis. Clinical characteristics of the patients are reported in Table 1.

**Table 1:** PSP and PD patients characteristics

	N.patients	Age (mean ± SD)	Gender	Disease duration at the evaluation	Disease stage	Treatment
PSP	50	69.82 ± 9.04 (range 51–105 years)	28 (56%) females	3.80 ± 2.06 years (range: 1–9 years)	Golbe Stage: stage 1: 8%; stage 2: 22%, stage 3: 32%; stage 4: 26%; stage 5: 12%	74% Levodopa 88% rasagiline (22% monotherapy) 56% amantadine
PD	100	69.19 ± 8.27 (range 51–89 years)	59 (59%) females	3.83 ± 2.25 years (range: 1–12 years)	H&Y stage: stage 1: 23%; stage 2: 43%; stage 3: 27%; stage 4: 7%	levodopa 61% rasagiline 22% (6 of them in monotherapy) Amantadine 3% Ropinirole 9% (2 of them in monotherapy)

Concerning the PSP rating scale, the mean value was 37.52 ± 15.69 (range 10–72), MoCA: 17.52 ± 6.32 (range 0–29); HAM-D: 8.80 ± 5.99 (range 1–24). Cognitive impairment was found in 92% and depressive symptoms in 48% of subjects.

Concerning the H&Y stage 23% of patients were in stage 1; 43% in stage 2, 27% in stage 3; and 7% in stage 4. Cognitive impairment was present in 11%, using MMSE^9^ and depressive manifestations in 39% of PD patients.^10^

### Frequency of non-motor symptoms

In the PSP group NMSS total domains score was 77.58 ± 42.95 (range 14–163).The most frequent NMS found in PSP was getting up regularly at night to pass urine (item of “Urinary symptoms” domain), present in 82% of subjects, followed by urinary frequency (“Urinary symptoms”) and fatigue (item of “Sleep/fatigue” domain), both with a prevalence of 68%.

In the PD group NMSS total score was 41.97 ± 35.45 (range: 0–215). The most frequent NMS in PD patients was fatigue (tiredness) or lack of energy (not slowness) (67%), followed by nocturia (61%) and urinary urgency (48%). The differences for these frequencies compared to the PSP group were significant for urinary urgency (*p* = 0.024) and nocturia (*p* = 0.01). Prevalence rates of NMS by NMSS domains are shown in Table 2.

**Table 2:** Prevalence of NMSS domains in PSP and PD patients

NMSS Domains	PSP (%)	PD (%)	P*
Cardiovascular/falls	50.0%	47.0%	0.733
Sleep/fatigue	92.0%	89.0%	0.774
Mood/apathy	88.0%	69.0%	0.015
Perceptual/hallucinations	36.0%	27.0%	0.264
Attention/memory	76.0%	59.0%	0.047
Gastrointestinal/tract	86.0%	71.0%	0.045
Urinary	92.0%	72.0%	0.005
Sexual function	18.0%	31.0%	0.117
Miscellaneous	56.0%	72.0%	0.066
NMS total	100.0%	99.0%	0.48

*Two-sample test of proportion

### Association of NMSS with other assessments

In the PSP group, analysis between rating scales and NMSS showed a high correlation between NMSS total and PSPRS total (*r*_S_ = 0.60; *p* < 0.01) and in NMSS and PSPRS domains, in particular the gastrointestinal tract had a high correlation with all the PSPRS domains: history (*r*_S_ = 0.48; *p* < 0.01), mentation (*r*_S_ = 0.47; *p* < 0.01), bulbar (*r*_S_ = 0.69; *p* < 0.01), ocular (*r*_S_ = 0.44; *p* < 0.01), gait (*r*_S_ = 0.42; *p* < 0.01), and total score (*r*_S_ = 0.55; *p* < 0.01).

Sleep and fatigue weremoderately correlated with some domains of the PSPRS: history (*r*_S_ = 0.45; *p* < 0.01), ocular (*r*_S_ = 0.46; *p* < 0.01), gait (*r*_S_ = 0.39; *p* < 0.01), and total score (*r*_S_ = 0.50; *p* < 0.01). A moderate correlation was also observed between NMSS mood/apathy and mentation (*r*_S_ = 0.43; *p* < 0.01), bulbar (*r*_S_ = 0.39; *p* < 0.01), gait (*r*_S_ = 0.37; *p* < 0.01) and total score (*r*_S_ = 0.43; *p* < 0.01).

The cognitive scale MoCA had a moderate correlation with NMSS attention/memory domain (*r*_S_ = 0.43; *p* < 0.01); the HAM-D was closely correlated with NMSS mood/apathy (*r*_S_ = 0.54; *p* < 0.01) and attention/memory domains (*r*_S_ = 0.41; both *p* < 0.01). Total NMSS scores showed a moderate correlation with cognition and depressive symptoms (0.46 for MoCA; 0.57 for HAM-D).

The analysis of NMSS domains demonstrated that the most severe domains in PSP are Urinary (mean 38.78), Gastrointestinal (mean 32.56) and Sleep/Fatigue (mean 27.50). The NMS burden in the PSP group was 4 (very severe).

### Findings in the PD group

In PD the most severe domains are Urinary (mean 20.47), Sleep/Fatigue (mean 16.25), and Gastrointestinal (mean 12.67). The NMS burden in the PD group was 3 (severe).

**Fig. 1:**
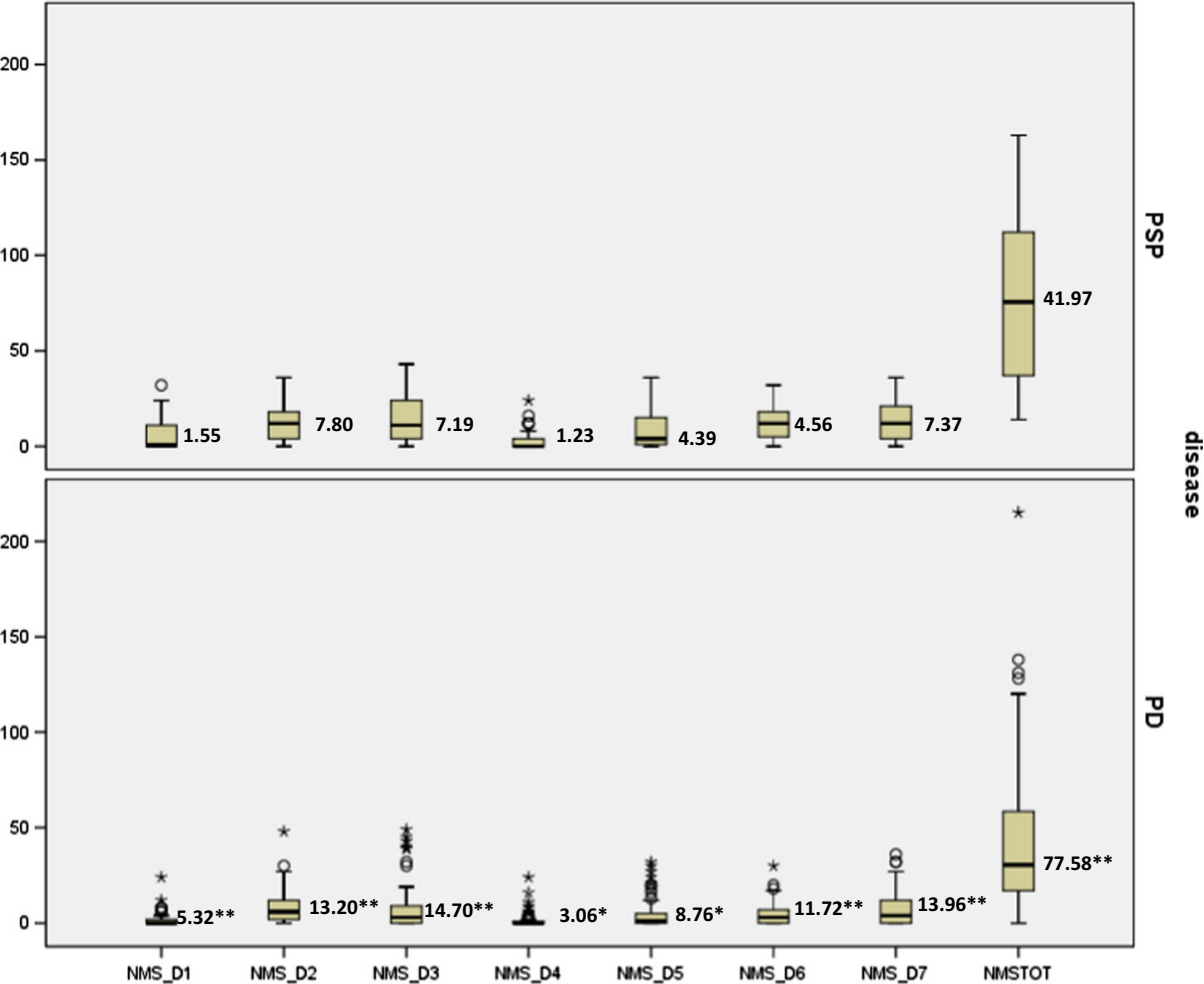
Severity of NMSS domains scores in PSP and PD patients. Only domains with significant differences between PSP and PD patients are presented. For differences between PSP and PD scores: Mann–Whitney test, *p < 0.05, **p < 0.001. NMS: Non-Motor Symptoms scale, D1: cardiovascular, D2: sleep/fatigue, D3: mood/apathy, D4: perceptual/hallucinations, D5: attention/memory, D6: gastrointestinal, D7: urinary, TOT: NMS total

The comparative analysis (Fig. 1) showed that NMS total score (*p* < 0.0001), sleep/fatigue (*p* = 0.0007), mood/apathy (*p* = 0.0001), gastrointestinal (*p* < 0.0001), and urinary dysfunction (*p* = 0.0001) domains were significantly more severe in PSP than in PD.

## Discussion

Currently, our study is one of the largest observational reports on NMS in PSP using a validated tool and classification system and highlights the frequency and severity of NMS in this population. We reported for the first time the very severe burden of NMS in PSP using the NMSS burden grading system and also confirmed that NMS are more frequent and severe in PSP than in PD. This fact is consistent with the progression and severity of this atypical parkinsonism. Our investigation has highlighted not only the frequency of each non-motor symptom (as probed by PRIAMO study) but also its severity using a comprehensive and global instrument, the NMSS.^11^

This study evidenced that urinary symptoms are the most frequent NMS domain associated to PSP, where “getting up regularly at night to pass urine” was reported by 82% of patients and “urinary frequency” by 50%. These data showed a significant difference with the PD group (*p* = 0.010 for the nocturia item and *p* = 0.024 for urinary frequency item). Urological dysfunction has rarely been studied in patients with PSP and there are few studies aimed to clarify their etiology.^12^

Together with comorbidities typical of age, detrusor hyperreflexia, detrusor sphincter dyssinergia and forebrain dysfunction seem to be related to these urinary disorders.^13^

Our study has highlighted how frequent such disorders are in PSP and how important it is to clarify the origin in order to define an effective treatment.

Gastrointestinal symptoms, particularly related to upper GI tract, were also very frequent: as expected, swallowing abnormalities were reported by 64% of PSP patients and by 24% of PD patients (*p* = <0.001). A recent survey on 35 PSP patients has shown that dysphagia is very frequent in the early stages of the disease and tends to involve the majority of subjects as disease progresses. The authors also highlight how, even though dysphagia is a recognized symptom of PSP, is often not investigated by specialists. They emphasize how addressing this problem is crucial to prevent aspiration pneumonia, which is the first cause of death in PSP patients. The present study demonstrates that NMSS can be used as an effective tool to detect early fundamental symptoms of the disease.^14^

Interestingly, we report that constipation was also more frequent in PSP patients (58 vs. 47%). This aspect will need further investigation.

The mean duration of PSP disease in our sample was 3.80 years vs. 3.83 years observed in the PD group, showing a trend that these urological and gastrointestinal dysfunction may occur earlier in PSP than in PD.

Significant difference was found in the item “changes in taste or smell” in the PD population (45%, *p* = 0.013), strengthening the hypothesis that hyposmia represents a risk factor for PD and could serve as a clinical differentiating marker between PSP and PD in some cases. In a recent meta-analysis on the prevalence of non-motor symptoms before and after the diagnosis, hyposmia was the most frequent non motor symptom observed with a frequency of 36% before diagnosis compared to 17% in the control group.^15^

The NMSS has also highlighted a higher score in the domains Mood/Cognition and Attention/Memory in PSP patients.

Most of PSP patients referred deficit in the “attention/memory” domain. These results correlate significantly correlate with the score obtained in the MoCA test: PSP patients tend to have extreme slowness in processing information, and dysfunctions in executive and visuospatial functions are confirmed in literature. Arena et al.^15^ found that literacy skill impairment (a category comprehending difficulties in reading, spelling, anomia, paraphasia, hesitancy of speech and difficulty understanding instruction) was as frequent as motor symptoms at the onset of the disease. These dysfunctions, as well as memory impairment, were not captured by the Mini Mental State Examination that authors used to assess enrolled subjects. MoCA has instead demonstrated to be efficacious in detecting early and subtle cognitive alterations and can be used as the first step of screening assessment in PSP patients.

Depression was present in 48% of subjects with PSP and 39% of PD patients. Regarding the NMSS, significant differences were found in various NMS sub-items: 48% of PSP patients referred a loss of interest in surrounding (PD patients 31%; *p* = 0.049); 54% difficulty experiencing pleasure (PD patients 27%, *p* = 0.002), 52% lack of motivation; 62% nervous feelings (PD patients 42%; *p* = 0.025) and 64% sad appearance (PD patients 41%; *p* = 0.009). The prevalence of more severe lack of motivation and apathy observed in PSP patients reflects degeneration in medial frontal regions and insular cortex.^16,17^

Since these symptoms have a negative impact on subjects and caregivers quality of life, it is essential to identify early and address them.

Even though sleep disorders have similar prevalence in PSP and PD patients, they resulted to be more burdensome in PSP patients (*p* < 0.001). The presence of greater axial stiffness, difficulties turning in bed at night and restless legs can account for that but degeneration of brainstem structures have also been hypothesized as cause of sleep disruption.^18^

The results of this study are in line with the founding of the NNPIPPS Study group,^6,7^ which showed higher rate of cognitive, behavioral and urinary dysfunctions in PSP patients.

The correlation analysis showed a significant association between PSP scale total score and NMSS total score and sub scores of some domains (“sleep/fatigue”, “mood/cognition”, “gastrointestinal tract”, “perceptual problems/hallucinations”), MOCA total score (inverse association) and HAM-D Total Score, as shown in Table 3. The differences of the severity of cognitive impairment and depression between PSP and PD patients are in line with the different disease progression.

**Table 3:** Correlation analysis

	NMS_D1	NMS_D2	NMS_D3	NMS_D4	NMS_D5	NMS_D6	NMS_D7	NMS_D8	NMS_D9	NMSTOT
Age	050	−061	−051	242	235	084	138	−143	030	094
psp_history	319*	450**	314*	481**	262	482**	200	−093	342*	540**
psp_mentation	297*	273	426**	252	455**	466**	233	137	117	599**
psp_bulbar	255	324*	395**	391**	113	695**	−009	073	216	506**
psp_ocular	077	456**	357*	290*	077	441**	−003	−005	327*	414**
psp_limb	201	248	166	384**	103	162	176	089	272	327*
psp_gait	360*	388**	371**	303*	020	417**	283*	046	139	466**
psp_total	323*	501**	434**	478**	210	554**	167	030	311*	602**
Moca_total	−338*	−213	−308*	−268	−430**	−289*	−234	072	−269	−456**
HMD_17_total	290	356*	537**	328*	412**	038	262	209	220	573**
HMD_21_total	311*	352*	528**	326*	431**	042	259	205	217	571**

*Note*: *p < 0.05 (Spearman’s rank)

** *p* < 0.01

This study has some limitations. The PSP group of patients were compared with PD subjects enrolled in a different study and evaluated with different scales (MoCA vs. MMSE, …); therefore a in-depth comparison between the two groups can result difficult. However, it has to be pointed out that the real intent of our study was to analyse the frequency and severity of NMS, which were investigated through the administration of the same validated scale in the two subsets of patients. An additional limitation is the low representation of patients in the most advanced stages of disease, which is more evident in the PD sample and can lead to an underestimation of NMS in this group, although this is a usual characteristic of the clinical samples. A careful matching between the two groups has been performed to ensure that populations were as homogeneous as possible, and to minimize the bias. Keeping these limitations in mind, the results of this study can represent an important contribution to the understanding of NMS in PSP.

NMS has emerged as one of the key unmet needs in neurodegenerative disorders such as PD. In the past decade the issue has been addressed by validation and widespread use of comprehensive tools such as the NMSS. In PD the NMS burden has one of most consistent and robust association with Qol.^19^ Nevertheless, NMSs continue to be an unmet need and the knowledge of holistic profile and impact of NMS in parkinsonian conditions such as PSPis even less clear. Our paper is thus an initial pathfinder and will help develop this important area of research in PSP and related conditions in future by larger comparative real-life studies.

It would be very interesting to further investigate NMS in larger populations of PD/PSP patients, in order to identify, which NMS are more frequent in the early stages of each disease. In case if this investigation would end with significant outcomes, the NMSS would be a useful tool for clinicians to better orient the diagnosis and schedule the best treatment.

Moreover, since the research is now focusing on disease-modifying therapies, making an early and correct diagnosis would be crucial.

## Methods

This was an observational, cross sectional, multicenter study, conducted in one site in Italy and in 15 European countries (The Non-Motor International Longitudinal Study—NILS, National Institute of Health Research, UK; UKCRN No: 10084). The study protocol was approved by the ethics committee of the coordinating center (Comitato Etico IRCCS San Raffaele Pisana, Rome, Italy) and by the reference local ethic committees of each of the participating sites. The study was undertaken in accordance with Good Clinical Practice and the provisions of the International Conference on Harmonization, with all patients providing written informed consent.

### Sample

Fifty consecutive PSP patients as per National Institute for Neurological Disorders and Society for PSP criteria,^8^ and 100 Parkinson’s disease (PD) patients as per the United Kingdom Parkinson’s Disease Society Brain Bank criteria were enrolled in the study.^20^ Data from PD patients from the NILS,^21^ in a proportion PSP/PD = 1/2, matched in age, sex, and disease duration were obtained for comparison.

### Assessments

Non-motor symptoms were assessed using the Non-Motor Symptom scale (NMSS) in PSP and PD patients. NMSS is a 30 items scale including 9 domains: cardiovascular including falls, sleep/fatigue, mood/apathy, perceptual problems, attention/memory, gastrointestinal, urinary, sexual function and miscellaneous. Each item evaluates separately frequency (scores 1–4) and severity (scores 0–3), with scores assigned by a trained rater through interview with the patient and/or caregiver. Total score for each domain is obtained by the sum of the corresponding item scores (frequency x severity, with a range from 0, not present, to 12, maximum frequency and severity), and for the total scale by the sum of all domains (range from 0 to 320) higher scores indicates higher frequency and severity of symptoms.

NMS Burden was calculated using burden grading cut-off scores.^22,23^

For PSP patients, clinical evaluations were performed using the Progressive Supranuclear Palsy Rating Scale (PSPRS). PSPRS comprising 28 items in six categories: daily activities (by history), behavior, bulbar, ocular motor, limb motor and gait/midline symptoms. Scores range from 0 to 100, each item graded 0–2 (six items) or 0–4 (22 items), higher scores indicating higher severity of symptoms.^24^

The Hoehn and Yahr (HY) classification was used to establish the staging of PD. The original 5-stage HY scale was used in this study.^25^

In the PSP group, the evaluation was completed with the Montreal Cognitive Assessment (MoCA),^26,27^ a 30-point test (normal ≥ 26/30) assessing several cognitive domains,and the Hamilton depression rating scale (HAM-D) (Sum the scores from the first 17 items. 0–7 = normal; 8–13 = mild depression; 14–18 = moderate depression; 19–22 = severe depression; >23 = very severe depression),^28^whereas PD patients were tested with the and the Hospital anxiety and depression scale–subscale depression (HADS-D); higher scores indicating greater likelihood of depression or anxiety.^10^ Mini Mental State Examination (normal ≥ 26/30)^9^ respectively.

### Data analysis

Descriptive statistics (percentage, central tendency and dispersion measures) were used as needed.

Data did not fit normal distribution (Kolmogorov–Smirnov test).

The prevalence of each non-motor symptom was expressed by the percentage of patients scoring 1 or more points in each item of the NMSS.

We calculated the mean of scores in each domain in both PSP and PD group.

To analyze the association between NMSS and PSP scale, MoCA, HAM-D, Spearman’s rank correlation coefficients were calculated because assumptions for the use of parametric correlation tests were not met. For this study, correlation coefficients 0.35 to 0.50 were considered moderate.

For the comparative analysis of NMSS between PSP and PD, chi-squared test, Mann–Whitney test and Fisher’s exact test were used. Box and whiskers plot, representing the median, inter-quartile range, minimum and maximum and outliers of the distribution, was designed to represent the differences in NMSS scores between PSP and PD patients.

In both groups, patients were classified as with or without cognitive impairment and with or without depression, using recognized cut-off points of the respective cognitive and depression scales.^9,10,26,28^

### Data availability

The authors declare that all other data supporting the findings of this study are available within the paper and its supplementary information files.
